# Diverse PD-1, CD163, and FOXP3 Profiles in Primary and Metastatic Microenvironments of Prostate Cancer

**DOI:** 10.32604/or.2025.068023

**Published:** 2025-10-22

**Authors:** Ana Clara Ciglioni Salustiano, Gabriela Barbosa, Rodolfo Borges dos Reis, Amílcar Castro de Mattos, Athanase Billis, Leonardo O. Reis

**Affiliations:** 1 UroScience, State University of Campinas, Unicamp, Campinas, 13083-875, Brazil; 2 Medicine School of Ribeirão Preto, University of São Paulo, Ribeirao Preto, 14049-900, Brazil; 3 ImmunOncology, Pontifical Catholic University of Campinas, PUC-Campinas, Campinas, 13034-685, Brazil; 4 INCT UroGen, National Institute of Science, Technology and Innovation in Genitourinary Cancer, Campinas, 13083-970, Brazil; 5 Pathology Department, Pontifical Catholic University of Campinas, PUC-Campinas, Campinas, 13034-685, Brazil; 6 Pathology Department, State University of Campinas, Unicamp, Campinas, 13083-888, Brazil

**Keywords:** Prostate cancer, metastasis, primary tumor, programmed cell death protein 1, cluster of differentiation 163, Forkhead box P3

## Abstract

**Objective:**

The tumor microenvironment plays a pivotal role in prostate cancer progression and may differ across metastatic sites. This study aimed to evaluate and compare the primary and metastatic prostate adenocarcinoma tumor microenvironment.

**Methods:**

A total of 27 formalin-fixed paraffin-embedded tissue samples derived from 17 patients diagnosed with prostate adenocarcinoma, including the primary tumors, and the corresponding metastatic lymphatic and hematogenous lesions from various anatomical sites. Immunohistochemical labeling was performed using antibodies against Cluster of Differentiation 3 epsilon chain (CD3e), CD8 alpha chain (CD8a), Cluster of Differentiation 68 (CD68), Cluster of Differentiation 163 (CD163), Forkhead box P3 (FOXP3), Cytotoxic T-Lymphocyte–Associated protein 4 (CTLA-4), B7 homolog 3 (B7-H3), Programmed cell death protein 1 (PD-1), and Marker of proliferation Ki-67 (Ki-67). Comparisons were made between primary and metastatic tumors to assess differences in immune cell infiltration, checkpoint expression, and proliferative indices.

**Results:**

Samples were classified into three groups: Primary Tumor n = 12, Lymphatic Metastasis n = 7, and Hematogenous Metastasis n = 10. FOXP3 (*p* = 0.0017) and CD163 (*p* = 0.0316) expression levels were significantly higher in the Hematogenous Metastasis compared to both the Primary Tumor and Lymphatic Metastasis. PD-1 showed a clear trend (*p* = 0.0577) toward higher levels in the Primary Tumor compared to both the Hematogenous Metastasis and Lymphatic Metastasis groups, suggesting distinct immunological landscapes depending on tumor location and progression.

**Conclusion:**

Diverse PD-1, CD163, and FOXP3 profiles were observed in primary and metastatic microenvironments of prostate cancer. These findings may contribute to the development of personalized therapeutic strategies and novel prognostic tools beyond conventional histological and TNM staging.

## 1 Introduction

Prostate cancer (PC) is the second most common type of cancer diagnosed in men, following lung cancer [1]. Although only 6% of men with PC present with metastatic disease at diagnosis, approximately 90% of those who die from the disease exhibit metastases at the time of death [2,3]. Beyond complications such as pathological fractures and spinal cord compression, often requiring radiation therapy or orthopedic surgery, metastasis signifies disease progression to an incurable and lethal stage. This not only worsens the prognosis but also reinforces metastasis as one of the greatest concerns in the context of treatment strategies [4].

The current staging protocol (e.g., TNM) is based only on the parameters of the tumor cell (cell-autonomous process) and does not consider that the evolution of cancer reflects complex cellular and molecular interactions between the tumor and the patient’s immune system [5]. The prostate is considered an immunocompetent organ, filled with a small number of scattered leukocytes, mainly stromal and intraepithelial T and B lymphocytes, macrophages, and mast cells. In abnormal conditions, such as benign prostatic hyperplasia, prostatitis, proliferative inflammatory atrophy, and adenocarcinomas, the density and composition of immune infiltrates are frequently altered in comparison to normal tissue. Notably, specific immune cell profiles have been associated with cancer progression and may constitute key components of the anti-tumor immune response [6].

In this context, while primary tumors often develop within an increasingly immunosuppressive tumor microenvironment (TME), metastatic dissemination forces tumor cells to abandon the immunosuppressive “bunker” of the primary TME and colonize distant tissues where immune evasion mechanisms and stromal support are not yet established. In these nascent sites, disseminated cells are transiently vulnerable to immune surveillance, creating a potential window for therapeutic intervention [7].

Therefore, identifying precise prognostic markers that predict metastatic potential is critical to refine treatment choices, particularly for focal therapies, and enhance patient outcomes. Mapping the immune landscape across according to the International Society of Urological Pathology (ISUP) grades and between primary and metastatic lesions could provide prognostic insights that complement conventional histological grading and TNM staging [8].

## 2 Methods

### 2.1 Patient Samples and Ethical Approval

We used archival tissue samples (n = 28) obtained from 17 patients diagnosed with prostate adenocarcinoma after informed consent, and ethics committee approval of Hospital das Clínicas da Faculdade de Medicina de Ribeirão Preto of the Universidade de São Paulo (HCFMRP/USP), CAAE: 85464824.7.0000.5440. Primary tumors were defined as the original neoplastic tissue arising in the prostate gland, including infiltrative tumor extensions into adjacent organs such as the seminal vesicle, rectum, and bladder. Samples from these sites were considered part of the primary tumor group only if no evidence of metastatic dissemination was present. Metastatic lesions were classified separately as lymphatic or hematogenous metastases.

### 2.2 Definition and Classification of Tumor Samples

Patients who had previously undergone radiotherapy were excluded from the study. All samples were collected during surgical procedures, and none of the patients had received chemotherapy at the time of collection. However, detailed data on other treatments were limited, and paired pre- and post-treatment samples were not available. These factors were considered during data interpretation. Importantly, all patients were at advanced stages of prostate cancer and receiving androgen deprivation therapy (ADT) at the time of sampling, consistent with the standard of care. Thus, the immune profiles observed likely reflect the effects of castration-level hormonal suppression.

### 2.3 Histopathological Evaluation

Hematoxylin and eosin-stained slides of primary tumors and metastases were reviewed and graded according to the International Society of Urological Pathology (ISUP) consensus system, which refines Gleason scoring into five prognostic grade groups (ISUP grades 1–5) to enhance prognostic stratification [9].

### 2.4 Tissue Processing and Immunohistochemistry

Paraffin-embedded tissue blocks were sectioned on a Biocut 1130 rotary microtome (Reichert-Jung, Munich, Germany). After deparaffinization, slides underwent our overnight immunohistochemistry protocol. Endogenous peroxidase was quenched with 10-volume hydrogen peroxide for 15 minutes, and antigen retrieval was carried out in either 0.01 mol/L citrate buffer (pH 6) or Tris-EDTA (pH 8.9), per antibody requirements (Supplementary Table S1).

To block nonspecific binding, sections were incubated in Molico® skim milk, dried, and outlined with a hydrophobic barrier. Primary antibodies targeting proliferation (Ki-67), T cells (CD3e, CD8a, FOXP3), macrophage polarization (CD68, CD163), and immune checkpoints (PD-1, CTLA-4, B7-H3) were diluted in 1% BSA and applied overnight at 4°C, then incubated for 1 hour at 37°C. The catalog number, dilution ratio, and manufacturer’s information for all antibodies are provided in Supplementary Table S1.

After washing, slides were incubated with the appropriate anti-mouse or anti-rabbit IgG secondary antibody (1:200 in 1% BSA) for 90 minutes at 37°C, as detailed in Supplementary Table S1. Finally, slides were counterstained with hematoxylin, mounted using Entelan® resin (Merck, Darmstadt, Germany), and coverslipped.

### 2.5 Image Acquisition and Scoring

Imaging was performed on a Carl Zeiss Axio-Imager® A1 microscope (Carl Zeiss, Oberkochen, Germany) using AxioVision® software (version 4.9.1). For each slide, five randomly selected high-power fields (40× magnification) were captured under consistent illumination settings. Staining intensity was scored semi-quantitatively on a four-point scale: 1 = negative staining, 2 = weakly positive, 3 = positive, and 4 = strongly positive. For each case, the modal value (i.e., the most frequently observed score across the five fields) was recorded as the representative score for that sample.

### 2.6 Data Aggregation and Quantitative Analysis

To evaluate group-level trends, median values of individual sample scores were calculated per immunomarker and stratified by tissue origin: Primary Tumor, Lymphatic Metastasis, or Hematogenous Metastasis. Although inspired by the traditional H-score method, this approach does not apply proportional weighting and should therefore be considered a modified semi-quantitative H-score.

### 2.7 Statistical Analysis

Statistical analyses were performed using the SAS System for Windows, version 9.4 (SAS Institute Inc., Cary, NC, USA), and R software (R Foundation for Statistical Computing, Vienna, Austria; version 4.3.1, https://www.R-project.org/). Descriptive measures were calculated to compare groups, including mean, standard deviation, minimum, maximum, and median values. Statistical comparisons were conducted using the Kruskal–Wallis test to assess differences in immunomarker expression across the three anatomical groups, followed by Dunn’s test for multiple comparisons when necessary. The significance level adopted for the study was 5% (*p* < 0.05) for all statistical analyses.

## 3 Results

**Figure 1:**
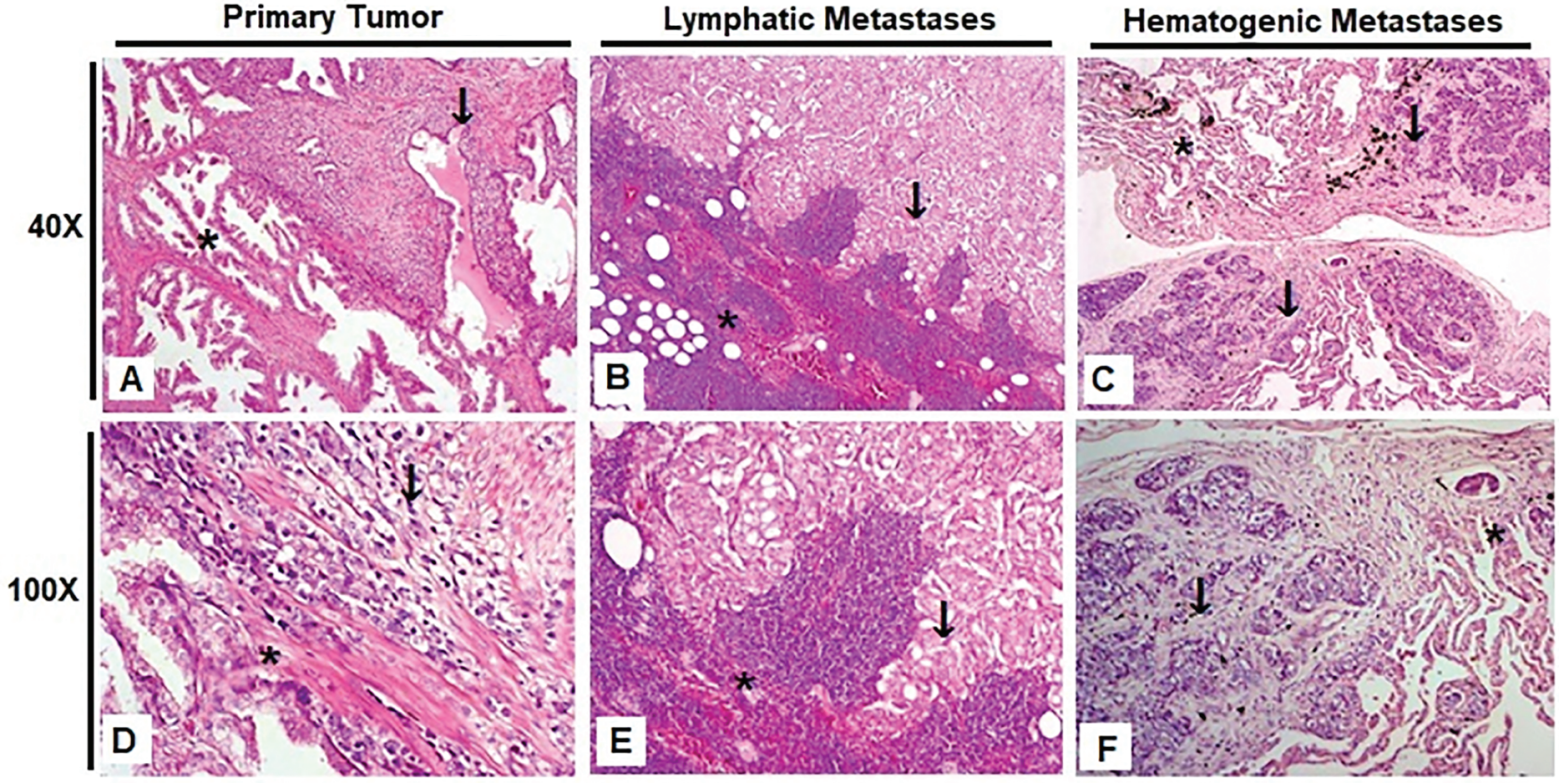
Representative hematoxylin and eosin (H&E) stained slides from the three experimental groups, photographed at 40× and 100× magnification. Scale bars: 200 µm (40×) and 50 µm (100×). (**A**,**D**) Prostate; (**B**,**E**) Obturated iliac lymph node; (**C**,**F**) Lung. Tumor regions are indicated by arrows, and adjacent non-neoplastic tissue is marked with asterisks

We obtained a total of 27 formalin-fixed paraffin-embedded tissue samples derived from 17 patients diagnosed with prostate adenocarcinoma. These samples included the primary tumors and the corresponding metastatic lymphatic and hematogenous lesions from various anatomical sites. Fig. 1 shows representative slides of H&E staining photographed at 40× and 100× magnification, highlighting areas of tumor and adjacent tissue. Sample origin and classification details are provided in Supplementary Tables S2 and S3.

The samples were categorized into three distinct groups based on their origin and metastatic status. The first group, Primary Tumor, consisted of 12 samples and included tissues other than the prostate itself, such as the seminal vesicle, rectum, and bladder, which were infiltrated by the primary tumor. The second group, Lymphatic Metastasis, comprised 7 samples representing prostate cancer that had spread to lymph nodes. Lastly, the third group, Hematogenous Metastasis, included 10 samples of prostate cancer metastases found in distant sites such as bone, lung, and bone marrow, indicating dissemination through the bloodstream.

**Figure 2:**
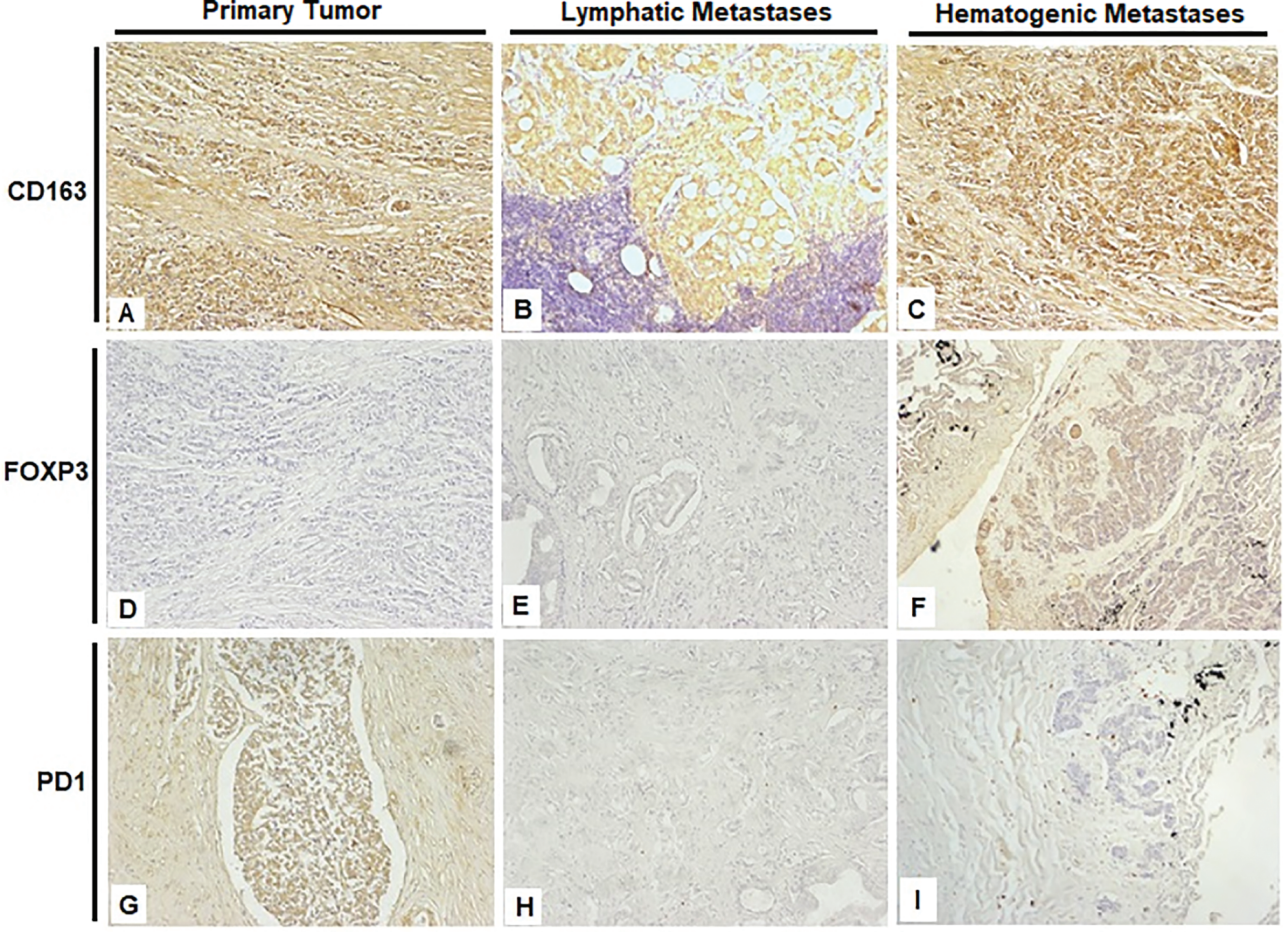
Representative immunohistochemical staining images from the three experimental groups, photographed at 40× magnification. (**A**,**D**,**G**) Prostate; (**B**,**E**,**H**) Obturated iliac lymph node; (**C**,**F**,**I**) Lung. Scale bar: 200 µm

The distribution of marker expression across groups is summarized in Supplementary Table S4. FOXP3 (*p* = 0.0017) and CD163 (*p* = 0.0316) expression levels were significantly higher in the Hematogenous Metastasis compared to both the Primary Tumor and Lymphatic Metastasis. PD-1 showed a clear trend (*p* = 0.0577) toward higher levels in the Primary Tumor compared to both the Hematogenous Metastasis and Lymphatic Metastasis groups, Fig. 2.

Supplementary Figure S1 demonstrates the distribution and localization of CD3e, CD8a, CD68, CTLA-4, B7-H3, and Ki-67 across different tissues in the three experimental groups. Complete descriptive statistics and *p*-values are presented in Supplementary Table S5.

The distribution of positive immune cells was generally sparse, with no predominant spatial pattern observed in relation to either the tumor or peritumoral compartments. There was no consistent clustering or exclusion of immune cell subsets such as PD-1^+^ cells, FOXP3^+^ Tregs, or CD163^+^ macrophages.

For the subset of primary tumor samples, we also investigated whether immunomarker expression varied according to the histological ISUP grading system; no statistically significant differences were found across ISUP grades 1 to 5 (data not shown).

## 4 Discussion

Our study demonstrates distinct immune profiles across primary tumors, lymphatic metastases, and hematogenous metastases in prostate cancer. Of the nine markers evaluated, FoxP3 and CD163 exhibited a significant increase in hematogenic metastasis compared to primary tumor and lymphatic metastasis, while PD-1 showed a clear trend to increase in the primary tumor compared to lymphatic and hematogenic metastasis.

FOXP3, a hallmark of regulatory T cells (Tregs), was elevated in both primary tumors and, most prominently, in hematogenous metastases, reflecting enhanced local immunosuppression and potentially worse prognosis. Tregs in the tumor microenvironment can generate an anti-tumor immune suppression response, favoring cancer progression [10].

CD163, indicative of M2-polarized macrophages, was also upregulated and correlates with tumor progression and metastasis; in bladder cancer, high CD163 expression predicts worse survival [11]. Notably, M2 macrophage infiltration has been linked to angiogenesis, suggesting a direct role in facilitating metastasis [12].

PD-1 is an immune checkpoint receptor expressed on activated T cells that plays a key role in downregulating anti-tumor immune responses. Its increased expression in lymphatic metastases suggests a more inflamed yet functionally exhausted microenvironment, potentially contributing to resistance to immunotherapy [13,14].

The higher expression of FOXP3 and CD163, specifically in the hematologic metastasis, suggests a more immunosuppressive tumor microenvironment at this stage of the disease, possibly reflecting greater infiltration of Tregs and M2-type macrophages, respectively. This observation is consistent with previous studies indicating the role of these cells in immune system escape and tumor progression in prostate cancer [15,16].

**Figure 3:**
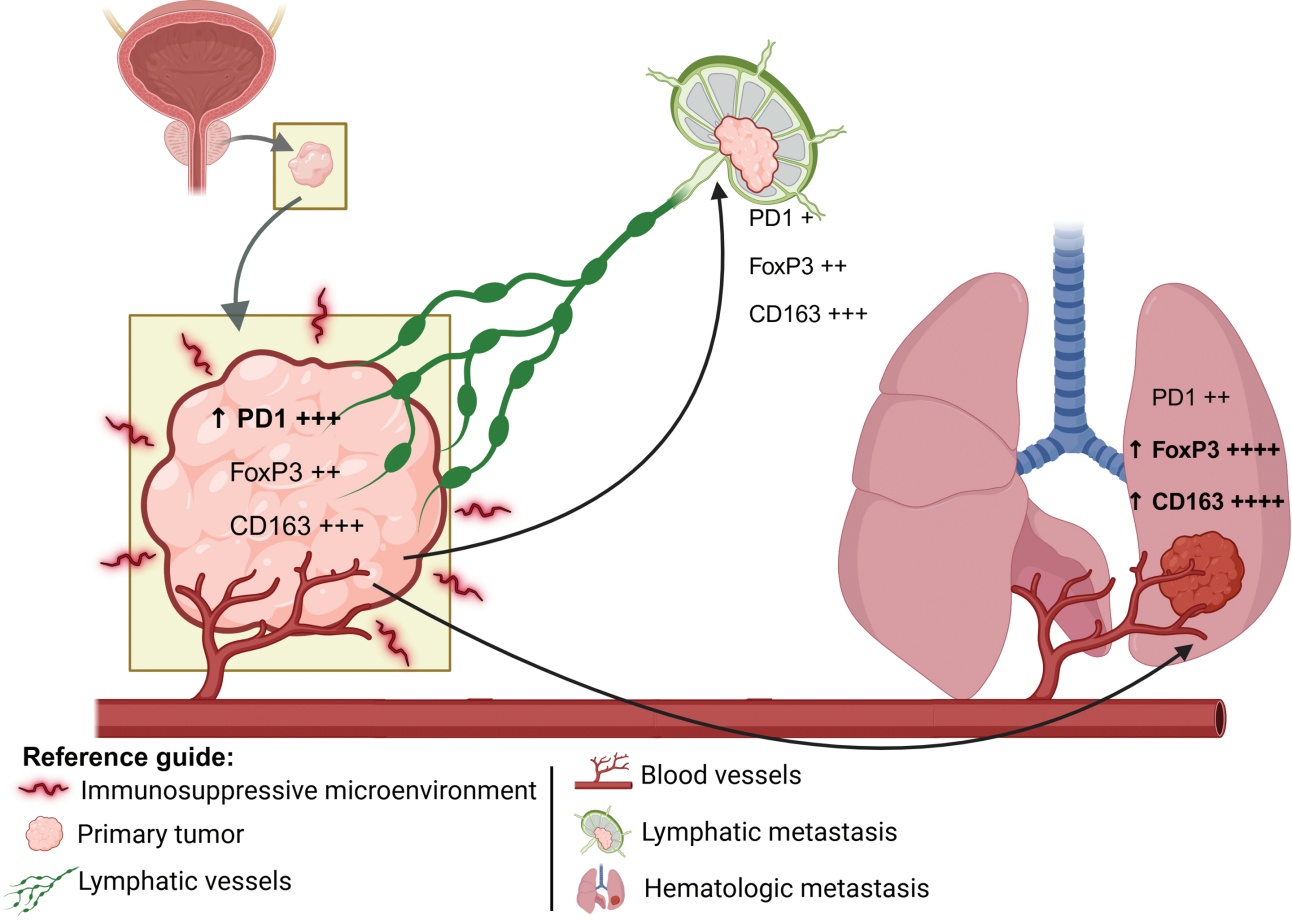
Schematic representation of the spatial progression of metastasis: the primary tumor represents an immunosuppressive environment; the lymphatic metastasis occurs closer to the primary site; and the hematogenous metastasis typically reaches more distant organs. The expression levels of PD1, FoxP3, and CD163 are represented semi-quantitatively, where “+” indicates low expression, “++” moderate expression, “+++” high expression, and “++++” very high expression. The upward arrow (↑) denotes a relative increase in expression compared to the previous metastatic site. Illustration created with BioRender.com

PD-1 expression, although with p slightly above the significance threshold, showed a pattern of variation between all groups, which may reflect the progressive involvement of T-cell exhaustion mechanisms during tumor evolution [17]. These observations are summarized in a conceptual model (Fig. 3), which illustrates the evolution of the tumor immune microenvironment from primary tumors to lymphatic and hematogenous metastases.

Interestingly, our findings also align with broader evidence suggesting that metastatic tumors, in general, exhibit fewer tumor-infiltrating lymphocytes (TIL) than primary tumors, creating a more immunologically inert milieu and potentially reducing the efficacy of checkpoint inhibitors in advanced stages [18,19]. Although some reports find comparable checkpoint expressions between primary and metastatic sites [18], others highlight significant heterogeneity, such as variable TIM-3 and PD-1 levels depending on tumor type and metastatic location [20]. In prostate cancer, primary tumors typically exhibit higher immune-checkpoint scores than early metastases, reflecting a more established immunosuppressive niche [21].

Supplementary Table S6 describes the potential roles and mechanisms of the selected immunomarkers in prostate cancer. The lack of significant differences in Ki-67, B7-H3, CD8a, CD68, CD3e, and CTLA-4 implies a more uniform presence of proliferative and cytotoxic populations, or perhaps their roles are less tightly linked to metastatic progression in our cohort. For example, Ki-67 has been described as a predictor of metastasis and mortality in treated prostate cancer [22], while B7-H3 expression correlates with disease progression [23]. The infiltration of CD8+ lymphocytes also serves as an independent prognostic factor [24], and immune cell infiltration in androgen-deprived prostate cancer has been characterized in detail [25]. Moreover, recent advances in T-cell engager therapy have opened new frontiers in prostate cancer immunotherapy [26]. These findings reinforce the prognostic potential of FOXP3, CD163, and PD-1 in characterizing the tumor immune microenvironment in metastatic prostate cancer.

Importantly, early metastatic niches appear more vulnerable than established primary tumors. Evidence indicates that the initial microenvironment is substantially distinct from the primary tumor, particularly in its lack of immediate immunosuppressive features—an aspect that may critically affect both tumor dynamics and the effectiveness of treatments across the progression of the disease [27,28].

In line with our findings, the primary tumor microenvironment develops progressively, fostering conditions that support neoplastic growth. In contrast, the initial metastatic sites present a less permissive environment, characterized by a distinct pattern of immunosuppressive cell recruitment [29]. Cancer-associated fibroblasts (CAFs) play a crucial role in the tumor microenvironment by promoting immunosuppression through extracellular matrix remodeling, cytokine secretion, and crosstalk with immune cells. In prostate cancer, CAFs support tumor progression and modulate the microenvironment partly via androgen receptor signaling, highlighting their importance as key stromal regulators contributing to tumor growth and therapy resistance [30].

Evidence in prostate cancer shows increased Treg infiltration in bone marrow metastases, promoting immune evasion [31], whereas primary tumors display elevated PD-1 on T cells, indicative of immune exhaustion [32]. Together, these differences underscore the metastatic microenvironment as both a therapeutic challenge and an opportunity—its nascent immunological state can be exploited by targeted strategies [33]. Moreover, as tumor cells disseminate, they exit the protective “bunker” of the primary niche and face heightened immune surveillance and stress, creating a transient window of vulnerability ripe for early intervention [7].

Heterogeneity in immune profiles between primary tumors and metastatic sites can profoundly affect treatment efficacy and drive resistance in advanced disease [34]. Although the extent of this heterogeneity remains debated [35], its impact on therapy selection is clear. Distinct anatomical sites can engage different immune-escape mechanisms, supporting the need for lesion-specific treatment strategies. Furthermore, primary tumors often exhibit greater immunogenicity than their metastatic counterparts, as reflected by higher scores on immunotherapy-predictive signatures [19].

Although our dataset does not include information on metastatic burden or progression-free survival, previous studies have reported associations between immune marker expression and advanced disease features in prostate cancer. High densities of FOXP3^+^ regulatory T cells have been linked to increased metastatic potential and poorer clinical outcomes [36,37]. Similarly, elevated infiltration by CD163^+^ M2 macrophages has been correlated with increased tumor aggressiveness and reduced progression-free survival, particularly in advanced and castration-resistant cases [38]. PD-1 expression on tumor-infiltrating lymphocytes has also been associated with higher Gleason scores and biochemical recurrence, suggesting its role in immune escape and disease progression [21].

In our cohort, we additionally examined whether immunomarker expression varied among primary tumors stratified by ISUP grade (1 to 5), and no statistically significant differences were observed. This absence of variation despite histopathological differences in the context of immunological diversity observed between primary tumor, lymphatic metastasis, and hematogenous metastasis supports the compartment hypothesis, in which the variability in the targeted immune markers appears to be driven more by differences in the tumor compartment than by variations of the primary tumor per se. These findings support the notion of a dynamic remodeling of the immune microenvironment along the metastatic cascade.

Moreover, evidence in advanced prostate cancer suggests that androgen receptor signaling contributes to the recruitment and polarization of immunosuppressive myeloid cells, including CD163^+^ macrophages, and enhances PD−1/PD-L1 immune checkpoint pathways, thereby reinforcing an immunosuppressive microenvironment that promotes therapeutic resistance in castration-resistant disease [39]. All patients in our cohort were receiving androgen deprivation therapy (ADT) with castration-level hormonal suppression at the time of sample collection, as per standard clinical management. This therapeutic context may have influenced the immune marker expression profiles observed, particularly those related to myeloid cell infiltration and checkpoint molecule upregulation. Notably, emerging evidence has expanded the scope of androgen receptor (AR)-mediated immune modulation beyond prostate cancer to other malignancies such as melanoma. In melanoma, AR activation has been linked to enhanced tumor invasiveness and impairment of critical immune cell functions, ultimately diminishing the efficacy of immune checkpoint therapies [40]. These observations underscore the potential for integrating AR-directed therapy with immune-targeted strategies in future cohort studies analyzing marker expression alongside clinical outcomes such as progression-free survival and metastatic burden.

The current study enhances our understanding of the tumor microenvironment across primary tumors, lymphatic metastases, and hematogenous metastases, suggesting that treatment strategies should be tailored to specific tumor sites due to distinct mechanisms and immune pathways. Although based on a small cohort (n = 17), the inclusion of 27 tumor samples from multiple metastatic sites provided consistent patterns, particularly through paired evaluations of primary and metastatic lesions, and highlights the potential for stage-specific therapeutic approaches.

In this context, it is important to recognize that organ-specific microenvironments can further influence immune cell infiltration at metastatic sites. Each tissue presents a unique set of biochemical cues, stromal architecture, and resident immune cells that modulate the recruitment and function of immune populations such as macrophages, regulatory T cells, and exhausted T lymphocytes [41]. For example, lung tissue often harbors a higher proportion of PD-1-expressing exhausted CD8+ T cells, contributing to localized immunosuppression, while other organs may promote alternative immunosuppressive mechanisms, such as Treg accumulation or M2 macrophage polarization [42].

These tissue-specific differences underscore the need for spatially informed interpretations of immune marker expression. However, due to the limited sample size in our study, we were not able to stratify the immune profiles by individual metastatic site. We acknowledge this limitation and highlight the importance of future prospective studies with larger, site-stratified cohorts to explore these mechanisms more deeply.

Study limitations: An important point to consider is the staging of the patients at the time of collection; many of the patients in our study already had metastatic disease in various tissues at the time the samples were taken, which may reflect different stages of tumor evolution compared to studies using only localized tumors or isolated metastases. Different treatments employed before collection or lack of treatment can also alter the expression of immune markers, such as PD-1, which is known to be induced by chronic tumor inflammation and chemotherapy [43]. The diversity of the metastatic sites analyzed (lymph nodes, bone marrow, lung, bladder) also imposes an additional degree of variability, as the local immune response can affect the infiltration and action of immune cells in different ways [44].

Another important limitation is the relatively small sample size, which restricted our ability to perform statistically robust subgroup analyses or establish reliable associations between immune marker expression and clinical or pathological features. Thus, the findings should be interpreted as descriptive and exploratory. It is important to highlight that, while the relatively small sample size represents a limitation, the present study provides exceptionally rare and valuable data by including matched samples of the primary tumor, lymphatic metastases, and hematogenous metastases—biological material that is seldom accessible, as these sites are not routinely biopsied or surgically removed in standard prostate cancer care. Our study’s rare dataset offers a unique opportunity to advance the biological understanding of metastatic progression while holding significant translational potential to inform future diagnostic strategies and guide the development of personalized therapeutic approaches in prostate cancer [45].

Recent studies have demonstrated that thermal ablation therapies, such as cryoablation, can modulate tumor-infiltrating immune cells by increasing CD8+ T cell infiltration and altering the CD4+/CD8+ ratio, potentially enhancing antitumor immunity. These immune changes reveal promising new targets for immunotherapy, suggesting that strategies aimed at boosting CD8+ T cell responses or modulating the CD4+/CD8+ balance could improve therapeutic outcomes [46]. Integrating such approaches with existing treatments may pave the way for innovative combinatorial therapies in cancer management.

Future studies should expand this analysis in larger, multicenter prospective cohorts to allow the comprehensive clinical annotation to further validate and expand upon our observations, as well as integrate spatial transcriptomics, multiplex imaging, and functional assays to validate the phenotypes and interactions suggested by the immunohistochemical data. Regarding the lack of a predominant spatial pattern observed in relation to either the tumor or peritumoral compartments, subsequent research employing double or triple staining methods, including multiplex immunofluorescence, would be valuable to uncover potential cell–cell interactions and their role in shaping immunosuppressive niches. Emerging spatially resolved technologies such as spatial transcriptomics, multiplex immunofluorescence, and spatial proteomics offer unprecedented resolution to map cellular phenotypes and interactions within their native tissue context. These approaches can elucidate the spatial organization of immune cell subsets, identify immunosuppressive niches, and reveal cell–cell communication networks that drive tumor progression and therapy resistance. Incorporating such advanced spatial analyses in future studies will be crucial to deepen our understanding of the immune microenvironment and to develop more precise, site-specific immunotherapeutic strategies in prostate cancer.

Additionally, characterizing the temporal evolution of the immune landscape—from localized to metastatic stages—may reveal critical windows of therapeutic opportunity, especially for immunotherapeutic interventions. Given the complexity and heterogeneity of metastatic progression, future studies with larger, site-stratified cohorts are essential to comprehensively understand the distinct immunosuppressive mechanisms and therapeutic vulnerabilities within different metastatic organ microenvironments. This approach will be pivotal for developing precise, site-specific immunotherapeutic strategies.

## 5 Conclusion

The findings of this study emphasize FOXP3, CD163, and PD-1 as key indicators of immune modulation across primary and metastatic prostate cancer. The dynamic shifts in immune landscapes, particularly the heightened immunosuppression in hematogenous metastases, underscore the need to tailor immunotherapeutic strategies by site and stage.

While lymphatic and hematogenous lesions share some features with primary tumors, their distinct immune contexts suggest different mechanisms of immune escape and therapeutic vulnerability. These findings reinforce the compartment hypothesis, in which variability in targeted marker expression is driven primarily by differences in the tumor microenvironment rather than by tissue type per se. Although limited by sample size and heterogeneity, our findings advocate larger, more diverse prospective cohorts to validate these markers and guide the development of precisely targeted interventions for metastatic prostate cancer.

## Supplementary Materials



## Data Availability

The authors confirm that the data supporting this study’s findings are available within the article or its Supplementary Materials.

## Supplementary Materials

The supplementary material is available online at https://www.techscience.com/doi/10.32604/or.2025.068023/s1.
