# Supplementary material for: Diverse PD-1, CD163, and FOXP3 Profiles in Primary and Metastatic Microenvironments of Prostate Cancer
Source: Oncol Res. 2025 Oct 22;33(11):3417–28. doi: 10.32604/or.2025.068023 (PMC12573194; doi:10.32604/or.2025.068023)
Supplement: Supplementary file 1 [file OncolRes-33-68023-s001.docx]

**Supplementary Table S1:** Information on antibodies used for immunohistochemistry analysis.

| **Primary Antibodies** | **Host Species** | **Dilution** | **Buffer Solution** | **Code** | **Source** | **Country** |
| --- | --- | --- | --- | --- | --- | --- |
| CD3e | Rabbit | 1:50 | EDTA | PA1-29547 | Invitrogen | USA |
| CD8a (AMC908) | Mouse | 1:50 | EDTA | 14-0008-82 | eBioscience | USA |
| CD68 | Mouse | 1:100 | Citrate | MA5-13324 | Invitrogen | USA |
| CD163 | Rabbit | 1:50 | Citrate | PA5-78961 | Invitrogen | USA |
| B7-H3 (anti-276) | Rabbit | 1:100 | Citrate | MA5-27898 | Invitrogen | USA |
| FOX-P3 (150D/E4) | Mouse | 1:100 | Citrate | 14-4774-82 | eBioscience | USA |
| CTLA-4 | Rabbit | 1:50 | EDTA | PA5-23967 | Invitrogen | USA |
| PD-1 (7A11B1) | Mouse | 1;50 | Citrate | MA5-15780 | Invitrogen | USA |
| Ki-67 | Rabbit | 1:100 | Citrate | Ab9260 | Merck | Germany |

**Supplementary Table S2:** Patient Samples and Corresponding Tissue Types in Prostate Cancer Study.

| **Patient** | **Sample** | **Tissue** |
| --- | --- | --- |
| **1** | 1 | Prostate |
| **1** | 2 | Prostate |
| **1** | 3 | Prostate |
| **1** | 4 | Lymphnode |
| **1** | 5 | Seminal Vesicle |
| **2** | 6 | Prostate |
| **2** | 7 | Lungs |
| **2** | 8 | Spine |
| **3** | 9 | Prostate |
| **3** | 10 | Obturated iliac lymphnode |
| **4** | 11 | Prostate |
| **4** | 12 | Bone Marrow |
| **5** | 13 | Prostate |
| **5** | 14 | Lymphnode |
| **6** | 15 | Obturated iliac lymphnode |
| **6** | 16 | Prostate |
| **7** | 17 | Femur |
| **8** | 18 | Right forearm |
| **9** | 19 | Pelvis |
| **10** | 20 | Bone Marrow |
| **11** | 21 | Bone Marrow |
| **12** | 22 | Obturated iliac lymphnode |
| **13** | 23 | Mediastinal lymphnode |
| **14** | 24 | Obturated iliac lymphnode |
| **15** | 25 | Lungs |
| **16** | 26 | Bladder |
| **16** | 27 | Bladder |
| **17** | 28 | Rectum |

**Supplementary Table S3:** Classification of Samples by Gleason Score.

| **Sample** | **Tissue** | **Pathology Report** |
| --- | --- | --- |
| **1** | Prostate | Usual acinar adenocarcinoma of the prostate, Gleason 4+5=9. ISUP Grade Group 5. |
| **3** | Prostate | Usual acinar adenocarcinoma of the prostate, Gleason 4+5=9. ISUP Grade Group 5. |
| **2** | Prostate | Usual acinar adenocarcinoma of the prostate, Gleason 4+5=9. ISUP Grade Group 5. |
| **5** | Seminal Vesicle | Seminal vesicles compromised by neoplasm in proximal portion, usual acinar adenocarcinoma of the prostate, Gleason 4+4=8. ISUP Grade Group 4. |
| **6** | Prostate | Usual acinar adenocarcinoma of the prostate, Gleason 4+5=9. ISUP Grade Group 5. |
| **9** | Prostate | Ductal carcinoma of the prostate, Gleason 4+5=9. ISUP Grade Group 5. |
| **11** | Prostate | Usual acinar adenocarcinoma of the prostate, Gleason 4+4=8. ISUP Grade Group 4. |
| **13** | Prostate | Adenocarcinoma of the prostate, Gleason 4+3=7 (grade 5 tertiary). ISUP Grade Group 3. |
| **16** | Prostate | Adenocarcinoma of the prostate, Gleason 4+3=7 ISUP Grade Group 3. |
| **27** | Bladder | Bladder infiltration of undifferentiated carcinoma of the prostate, usual acinar adenocarcinoma of the prostate, Gleason 4+5=9. ISUP Grade Group 5. |
| **26** | Bladder | Bladder infiltration of undifferentiated carcinoma of the prostate, usual acinar adenocarcinoma of the prostate, Gleason 4+5=9. ISUP Grade Group 4. |
| **28** | Rectum | Immunohistochemistry consistent with primary prostate neoplasia, infiltrating the rectum. Usual acinar adenocarcinoma of the prostate, Gleason 3+4=7. ISUP Grade Group 2. |

Note: ISUP, International Society of Urological Pathology.

**Supplementary Table S4:** Median Values of Immunomarkers in Primary Tumor, Lymphatic Metastasis, and Hematogenous Metastasis Groups.

| **Median** | **Primary Tumor** | **Lymphatic Metastasis** | **Hematogenous Metastasis** |
| --- | --- | --- | --- |
| **Ki-67** | 3.0 (1.0-4.0) | 3.0 (1.0-4.0) | 2.5 (1.0-4.0) |
| **CTLA4** | 4.0 (4.0-4.0) | 4.0 (3.0-4.0) | 4.0 (4.0-4.0) |
| **FOXP3** | 2.0 (1.0-4.0) | 2.0 (2.0-3.0) | 4.0 (2.0-4.0) |
| **B7-H3** | 3.5 (3.0-4.0) | 4.0 (3.0-4.0) | 3.0 (2.0-4.0) |
| **CD8a** | 2.0 (2.0-3.0) | 2.0 (2.0-3.0) | 2.0 (2.0-3.0) |
| **PD1** | 3.0 (1.0-4.0) | 1.0 (1.0-4.0) | 2.0 (1.0-2.0) |
| **CD68** | 1.0 (1.0-3.0) | 1.0 (1.0-1.0) | 1.0 (1.0-2.0) |
| **CD163** | 3.0 (2.0-4.0) | 3.0 (2.0-4.0) | 4.0 (3.0-4.0) |
| **CD3e** | 3.0 (2.0-4.0) | 3.0 (2.0-4.0) | 3.5 (2.0-4.0) |

**Supplementary Table S5:** Comparison between groups by Immunomarker.

| **Primary Tumor Lymphatic Meta Hematogenous Meta Total**  **Variable (N=12) (N=7) (N=10) (N=29)       P-Value** |
| --- |
| H-score Ki-67  (Mean ± SD (N))    2.8 ± 1.2 (N=12)      2.9 ± 1.3 (N=7)     2.4 ± 1.3 (N=10)     2.7 ± 1.3 (N=29)   0.6742¹  (Median (min-max) 3.0 (1.0-4.0)         3.0 (1.0-4.0)       2.5 (1.0-4.0)        3.0 (1.0-4.0)  H-score CTLA4   (Mean ± SD (N))    4.0 ± 0.0 (N=10)      3.7 ± 0.5 (N=7)     4.0 ± 0.0 (N=9)      3.9 ± 0.3 (N=26)   -   (Median (min-max) 4.0 (4.0-4.0)         4.0 (3.0-4.0)       4.0 (4.0-4.0)        4.0 (3.0-4.0)  H-score **FOXP3**  **(Mean ± SD (N)    2.2 ± 0.9 (N=11)      2.1 ± 0.4 (N=7)     3.5 ± 0.7 (N=10)     2.6 ± 1.0 (N=28)   0.0017¹***  **(Median (min-max) 2.0 (1.0-4.0)         2.0 (2.0-3.0)       4.0 (2.0-4.0)        2.0 (1.0-4.0)**  H-score B7-H3   (Mean ± SD (N))    3.5 ± 0.5 (N=10)      3.6 ± 0.5 (N=7)     3.1 ± 0.9 (N=9)      3.4 ± 0.7 (N=26)   0.5289¹   (Median (min-max) 3.5 (3.0-4.0)         4.0 (3.0-4.0)       3.0 (2.0-4.0)        3.5 (2.0-4.0)  H-score CD8a   (Mean ± SD (N))     2.4 ± 0.5 (N=11)     2.1 ± 0.4 (N=7)     2.3 ± 0.5 (N=9)      2.3 ± 0.5 (N=27)   0.5920¹   (Median (min-max)  2.0 (2.0-3.0)        2.0 (2.0-3.0)       2.0 (2.0-3.0)        2.0 (2.0-3.0)  H-score **PD1**  **(Mean ± SD (N))     2.5 ± 0.9 (N=12)     1.7 ± 1.1 (N=7)     1.8 ± 0.4 (N=10)     2.1 ± 0.9 (N=29)   0.0577¹**  **(Median (min-max)  3.0 (1.0-4.0)        1.0 (1.0-4.0)       2.0 (1.0-2.0)        2.0 (1.0-4.0)**  H-score CD68   (Mean ± SD (N))     1.3 ± 0.7 (N=10)     1.0 ± 0.0 (N=6)     1.1 ± 0.3 (N=9)      1.2 ± 0.5 (N=25)   -   (Median (min-max) 1.0 (1.0-3.0)        1.0 (1.0-1.0)       1.0 (1.0-2.0)        1.0 (1.0-3.0)  H-score **CD163**  **(Mean ± SD (N))     3.1 ± 0.7 (N=11)     2.9 ± 0.7 (N=7)     3.8 ± 0.5 (N=8)      3.2 ± 0.7 (N=26)   0.0316¹**  **(Median (min-max)   3.0 (2.0-4.0)        3.0 (2.0-4.0)       4.0 (3.0-4.0)        3.0 (2.0-4.0)**  H-score CD3e   (Mean ± SD (N))     3.2 ± 0.6 (N=11)     3.2 ± 0.8 (N=6)     3.2 ± 0.9 (N=10)     3.2 ± 0.7 (N=27)   0.9706¹   (Median (min-max)  3.0 (2.0-4.0)        3.0 (2.0-4.0)       3.5 (2.0-4.0)        3.0 (2.0-4.0) |

Note: ¹Kruskal-Wallis test. *Multiple comparison (Dunn's test): Hematological Meta Group differs from the others.

**Supplementary Table S6:** Functional Roles and Mechanisms of Selected Immunomarkers in Prostate Cancer.

| **Immunomarker** | **Function in Prostate Cancer** | **Biological Mechanism** | **References** |
| --- | --- | --- | --- |
| **FOXP3** | Suppression of antitumor immune response | Transcription factor of regulatory T cells (Tregs) that suppress effector immunity | [10] |
| **PD-1** | Suppression of adaptive immune response | Checkpoint receptor that promotes T cell exhaustion | [14] |
| **CTLA-4** | Inhibition of antitumor immune response | Checkpoint receptor that blocks T cell activation | [17] |
| **CD163** | Polarization of macrophages toward M2 phenotype | M2 macrophages promote tumor progression and immune evasion | [16] |
| **Ki-67** | Indicator of cell proliferation | Nuclear expression in actively dividing cells | [22] |
| **B7-H3** | Coinhibition of T cell activation | Immune ligand that contributes to tumor immune evasion | [23] |
| **CD8a** | Cytotoxic activity against tumor cells | CD8+ T cells recognize and kill tumor cells presenting antigens | [24] |
| **CD68** | Presence of macrophages in the tumor | Marker of total macrophages in the tumor microenvironment | [25] |
| **CD3e** | Presence of total T cells | Component of the T cell receptor (TCR) complex in mature T cells | [26] |


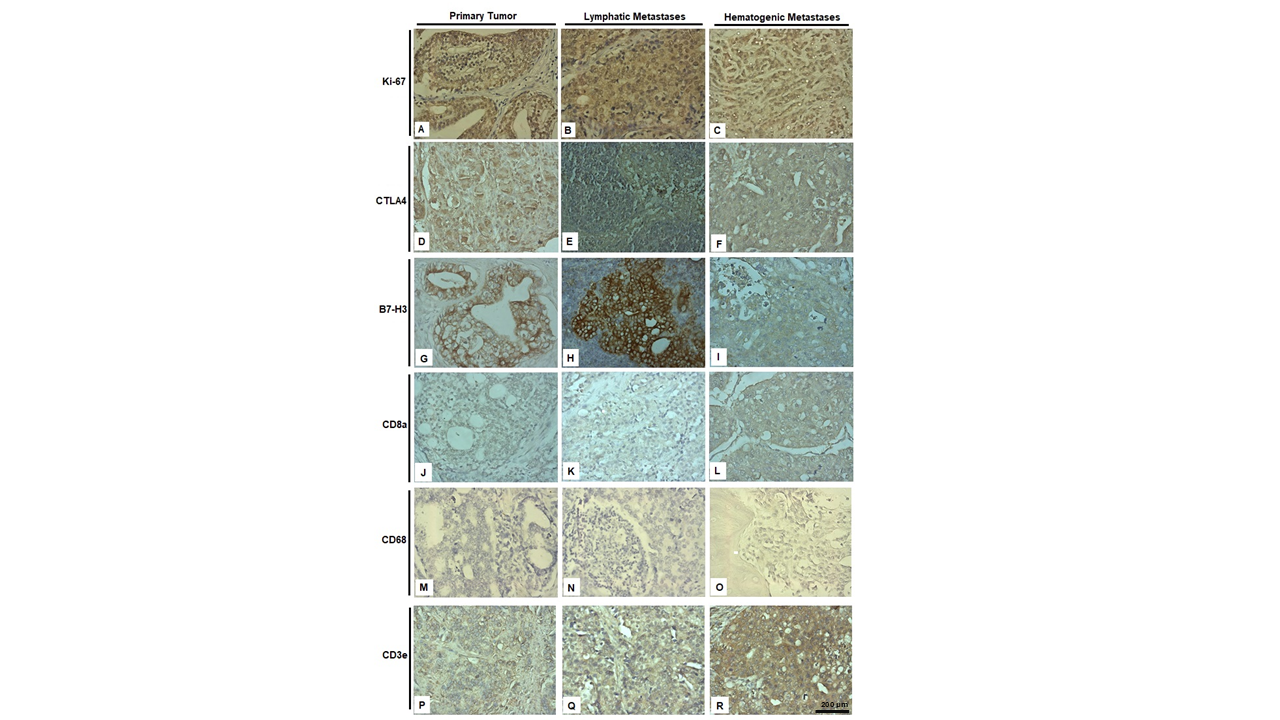


**Supplementary Figure S1:** Representative slides from the 3 groups, from the immunohistochemical analyses, photographed at 40× magnification. (**A**,**G**,**J**,**M**,**P**): Prostate. (**B**,**E**,**H**,**K**,**N**,**Q**): Obturated iliac lymph node. (**F**,**I**,**L**,**R**): Lung. (**C**,**O**): Bone marrow. (**D**): Seminal vesicles affected by usual acinar adenocarcinoma of the prostate in the proximal portion. Scale bar: 200 µm.
